# Safety and efficacy of completely transthoracic echocardiography guided leadless pacemaker implantation assisted by Panna guide wire: initial clinical experience

**DOI:** 10.3389/fcvm.2026.1791724

**Published:** 2026-03-26

**Authors:** Qiuzhe Guo, Zhiling Luo, Yulong Guo, Jinrui Guo, Chen Liu, Tao Guo, Xiangbin Pan

**Affiliations:** 1 Department of Ultrasonography, Fuwai Yunnan Hospital, Chinese Academy of Medical Sciences/Affiliated Cardiovascular Hospital of Kunming Medical University, Kunming, China; 2 Department of Electrophysiology, Fuwai Yunnan Hospital, Chinese Academy of Medical Sciences/Affiliated Cardiovascular Hospital of Kunming Medical University, Kunming, China; 3 Department of Structural Heart Disease, Fuwai Yunnan Hospital, Chinese Academy of Medical Sciences/Affiliated Cardiovascular Hospital of Kunming Medical University, Kunming, China; 4 Department of Structural Heart Disease, National Center for Cardiovascular Disease, State Key Laboratory of Cardiovascular Disease, Fuwai Hospital, Chinese Academy of Medical Sciences, Peking Union Medical College, National Clinical Research Center for Cardiovascular Diseases, Beijing, China

**Keywords:** echo-guided wire, fluoroscopy-free implantation, leadless pacemaker, Panna guidewire, transthoracic echocardiography

## Abstract

**Background and objective:**

Conventional leadless pacemaker (LP) implantation relies on fluoroscopy, exposing patients and operators to ionizing radiation and contrast-related risks. Transthoracic echocardiography (TTE) is a radiation-free alternative, but complete TTE-guided LP implantation remains challenging due to poor ultrasound visibility of interventional devices. This study evaluated the short-term safety, technical feasibility, and procedural efficiency of completely TTE-guided LP implantation assisted by the ultrasound-optimized Panna guidewire.

**Methods:**

This study utilized a prospectively protocolized, single-arm design for the TTE-guided cohort, with a retrospective comparative analysis against a historical fluoroscopy-guided control group. All safety and efficacy endpoints were formally predefined prior to patient enrollment. A total of 32 consecutive patients with LP implantation indications were screened during the study period (July 2024–July 2025), and 10 eligible patients underwent fluoroscopy/contrast-free, TTE-guided LP implantation using the Panna guidewire. Preoperative TTE acoustic window grading was performed, and standardized protocols (semi-quantitative “gooseneck” sign assessment, TTE-guided tug test) were applied during the procedure. A historical control group of 44 fluoroscopy-guided LP patients (January 2020–December 2023) was included, with propensity score overlap weighting-based comparative statistical analyses performed to balance baseline covariates and assess between-group differences. Procedural feasibility, short-term safety, pacing parameters, and skin-to-skin procedural duration were evaluated intraoperatively and during follow-up.

**Results:**

All 10 patients had optimal TTE acoustic windows (Grade 1). Procedural success was 100%, with no adverse events (median follow-up: 4.7 months) and stable device performance. Sensitivity analysis showed the TTE technique's effectiveness was not affected by operator experience. Compared with 44 propensity score-weighted controls, TTE-guided implantation had slightly longer but comparable procedural duration (62.78 ± 13.05 vs. 60.5 ± 19.1 min, *P* > 0.05) and comparable efficiency, eliminating radiation/contrast-related risks for high-risk patients (e.g., CKD, radiation sensitivity). Long-term follow-up (12/24 months) is ongoing per schedule.

**Conclusions:**

This preliminary experience demonstrates the short-term safety and technical feasibility of completely TTE-guided LP implantation assisted by the Panna guidewire, which eliminates radiation/contrast risks while matching fluoroscopy-guided efficiency. As a hypothesis-generating proof-of-concept study (small sample, incomplete long-term follow-up), these findings require validation in larger multicenter registries (*n* ≥ 50) with ≥24-month follow-up to confirm long-term safety and generalizability.

## 1 Introduction

Bradyarrhythmias are age-related common cardiovascular conditions (1%–3% of adults ≥65 years, up to 8% of those ≥80 years) (1, 2), arising from impaired cardiac electrical impulse generation or conduction and leading to significant morbidity and mortality (3, 4). Cardiac pacing remains the cornerstone of symptomatic bradyarrhythmia management, restoring physiological rhythm and improving outcomes (5, 6)—underscoring the need to advance pacing technologies for safety and precision, especially in vulnerable elderly and comorbid populations.

Compared with traditional transvenous pacemakers (TVPMs)—which use transvenous leads and a subcutaneous pulse generator pocket—leadless pacemakers (LPs) have emerged as a transformative right ventricular (RV) pacing modality over the past decade (7, 8). A key advantage is eliminating complications linked to transvenous leads and subcutaneous pockets, which account for 20%–30% of adverse events in TVPM patients (9, 10). Specifically, LPs reduce the risk of lead dislodgement (1%–5% incidence in TVPMs), pocket infection (0.5%–2% per year), and central venous thrombosis (3%–10% within 1 year) (11–13). LPs also have shorter procedural times (30–45 vs. 60–90 min median) and faster recovery (1–2 vs. 3–5 days hospital stay) (14, 15). These well-documented benefits have driven rapid clinical adoption of LPs as a key option for bradyarrhythmia management—especially in patients at high risk of lead- or pocket-related complications (16, 17).

Conventionally, LP implantation uses fluoroscopic guidance—the gold standard for visualizing vascular access, device delivery, and final positioning (18). However, this approach has inherent iatrogenic risks, limiting its use in vulnerable populations: First, ionizing radiation poses long-term risks: cumulative fluoroscopic doses increase patients’ cancer risk by 10%–20%, and medical staff (notably interventional cardiologists) face chronic occupational exposure (19, 20). Second, contrast agents (used to enhance vascular/cardiac visualization) cause contrast-induced nephropathy (CIN) in 2%–5% of general patients and 20% of those with preexisting CKD—prevalent in 30%–40% of elderly bradyarrhythmia patients (21, 22). Third, fluoroscopy's 2D imaging causes imprecise RV septal LP positioning—even experts may place devices on the RV free wall. This mispositioning raises long-term ventricular dysfunction, heart failure hospitalization, and mortality risks by disrupting electrical synchrony (23–25). As recently highlighted by (26), traditional fluoroscopy lacks the ability to visualize soft-tissue structures like the tricuspid valve and chordae tendineae, potentially leading to subclinical mechanical trauma during sheath advancement and device delivery. These linked limitations have fueled interest in alternative imaging modalities for safer, more precise LP implantation without radiation/contrast risks.

Echocardiography—specifically transthoracic echocardiography (TTE)—is a promising modality for guiding structural heart interventions, including LP implantation (27, 28). TTE offers unique advantages: non-ionizing ultrasound (no radiation), no contrast agents (avoiding CIN), and real-time high-resolution 2D/3D visualization of cardiac structures—including the RV septum (optimal pacing site), tricuspid valve, and surrounding myocardium (29, 30). Despite these benefits, earlier TTE-guided LP attempts faced two interconnected barriers to widespread adoption: (1) a lengthy operator learning curve (needing interventional skills and real-time ultrasound interpretation) (31, 32); (2) historical lack of ultrasound-optimized interventional equipment—standard guidewires/sheaths are hypoechoic under 2D TTE, causing frequent “tip loss” (poor device tracking), procedural delays, and higher iatrogenic injury risk (33–36). To date, few peer-reviewed reports document completely TTE-guided LP implantation without fluoroscopic backup, leaving a critical gap in radiation-free pacing therapy literature.

The Panna guidewire addresses the unmet need for ultrasound-optimized interventional equipment (37). Not a first-in-clinic device, it was previously validated in a 100-patient randomized controlled trial (RCT) for atrial septal defect (ASD) closure (38). This RCT showed complete procedural success, no device-related complications (e.g., guidewire-induced vascular injury, residual shunt), and markedly better ultrasound visibility than standard guidewires. Leveraging this clinical evidence, the Panna guidewire's core design—echo-enhancing nitinol spindle tip (0.8 mm diameter) and 0.035-inch hydrophilic shaft—is expected to overcome poor guidewire detectability in TTE-guided LP implantation. Specifically, its spindle tip generates a strong ultrasound signal, enabling real-time tracking even in suboptimal acoustic windows, while the hydrophilic coating reduces friction during vascular access and device delivery (37, 38).

Building on this innovation, completely TTE-guided LP implantation with the Panna guidewire is now underway at three Chinese clinical centers. Herein, we present initial single-center cohort results to explore the short-term safety (intraoperative and 3-month postprocedural complications) and technical feasibility (success rate, pacing parameters, procedural efficiency) of this fluoroscopy-free procedure, and describe a standardized TTE-guided protocol to improve reproducibility. This study's core innovations and contributions are threefold: First, to our knowledge, this is one of the early prospective, peer-reviewed reports describing a completely fluoroscopy-free, TTE-guided LP implantation technique, specifically utilizing an ultrasound-optimized guidewire (Panna), filling the critical gap in radiation-free pacing for patients with fluoroscopy/contrast contraindications. Second, we validate the Panna guidewire's cross-procedural versatility, extending its use from ASD closure to LP implantation and confirming its potential as a universal ultrasound-optimized tool for structural heart interventions. Third, we establish a standardized TTE workflow (ultrasound view selection, guidewire tracking, LP positioning) that mitigates the historical TTE-guided learning curve, laying a foundation for multicenter reproducibility. Collectively, this study provides preliminary evidence for a safer, more accessible LP implantation approach, benefiting high-risk subgroups (advanced CKD, pregnancy, hereditary radiation sensitivity). Future validation directions are outlined in the discussion.

## 2 Materials and methods

### 2.1 Study design

This study utilized a prospectively protocolized, single-arm design for the TTE-guided cohort, with a retrospective comparative analysis against a historical fluoroscopy-guided control group. All safety and efficacy endpoints were formally predefined prior to patient enrollment. Consecutive patients undergoing completely TTE-guided LP implantation at Fuwai Yunnan Hospital (Kunming, Yunnan, China) between July 2024 and July 2025 were prospectively enrolled per predefined inclusion/exclusion criteria. Written informed consent was obtained from all patients preoperatively to disclose the innovative nature of the TTE-guided technique and potential risks, consistent with ethical guidelines for clinical research involving novel interventions. Procedural and follow-up data were prospectively collected, and retrospective analysis was performed after completion of patient enrollment. The study was approved by the institutional review board (IRB) (No.:2024-041-02) of the hospital and complied with the Declaration of Helsinki. This study was reported in accordance with the Strengthening the Reporting of Observational Studies in Epidemiology (STROBE) statement (39). A historical control group of 44 patients who underwent fluoroscopy-guided LP implantation (January 2020–December 2023) at the same institution was included for comparative analysis (IRB Approval No.: 2024-081-01) (40). Propensity score overlap weighting was applied to minimize indication bias.

During the study period, 32 patients were screened for LP implantation. Of these, 22 patients were excluded: 8 due to inadequate TTE acoustic windows (Grade 3: failure to clearly visualize key structures even after optimized positioning); 3 due to severe tricuspid regurgitation or mechanical valves; 4 due to severe right ventricular abnormalities; 2 due to anticoagulation contraindications; 3 with expected survival <6 months; and 2 who refused informed consent. Ultimately, 10 patients were enrolled. (Please refer to Supplementary Figure S1 for the patient screening flowchart).

Inclusion criteria: (1) Adult patients (≥18 years) diagnosed with symptomatic bradyarrhythmia (e.g., sick sinus syndrome, atrioventricular block) meeting current VVI pacing indications; (2) Adequate vascular access (femoral vein diameter ≥6 mm confirmed by preoperative TTE); (3) Optimal TTE acoustic window [Grade 1: clear visualization of the right ventricular (RV) septum, tricuspid valve, and catheter/guidewire during pre-procedural assessment].

Exclusion criteria: (1) Contraindications to LP implantation (e.g., severe RV hypoplasia, intracardiac thrombus); (2) Allergy to materials of the Panna guidewire or Micra device; (3) Pregnancy or lactation; (4) Severe hepatic/renal dysfunction (eGFR <30 mL/min/1.73 m^2^); (5) Expected survival <12 months; (6) Participation in other interventional clinical trials within 3 months; (7) Suboptimal TTE acoustic window [Grade 3: failure to visualize target cardiac structures even after body position adjustment (left lateral decubitus+forward leaning position)].

To contextualize the current findings, a consecutive historical control group of 44 patients (January 2020–December 2023) who underwent standard fluoroscopy-guided LP implantation at the same institution was retrospectively identified. Propensity score overlap weighting was applied to minimize indication bias. Baseline characteristics and perioperative metrics were compared between the control and TTE-guided cohorts to evaluate relative safety, efficiency, and radiation exposure.

### 2.2 Definitions and end-points

All efficacy and safety endpoints were adapted from contemporary guidelines and large-device registries to ensure external validity (41–44) and were prospectively embedded in the electronic case-report form before database lock.

Procedural success: (i) successful femoral access without ultrasound-to-fluoroscopy conversion, (ii) deployment of the LP at the intended RV septal site, (iii) electrical parameters meeting Micra manual thresholds (R-wave ≥ 5 mV, impedance 300–1,500 Ω, capture ≤ 2.0 V @ 0.4 ms) (40), and (iv) absence of acute major complications (cardiac perforation, device dislodgement, vascular injury requiring intervention, or pericardial effusion >10 mm).

Adverse events: (i) major events, any of the following events documented at implant, 24 h, 1-, 3- and 6-month visits: death (any cause), cardiac perforation or tamponade, device dislodgement requiring repositioning, major bleeding (BARC ≥ 3b) (42), system-related infection (endocarditis, pocket or bloodstream), or vascular complication requiring surgery or transfusion (43.(ii) minor events, documented at the same time-points: groin haematoma > 5 cm or requiring compression, arteriovenous fistula < 3 mm, pseudoaneurysm (any size), asymptomatic pericardial effusion ≥ 5 mm on TTE, puncture-site thrombosis, self-limiting vasovagal episode, or any arrhythmia requiring intervention (44).

All events were adjudicated by two independent cardiologists blinded to imaging modality; disagreements were resolved by a third senior investigator.

### 2.3 Panna guidewire design

**Figure 1:**
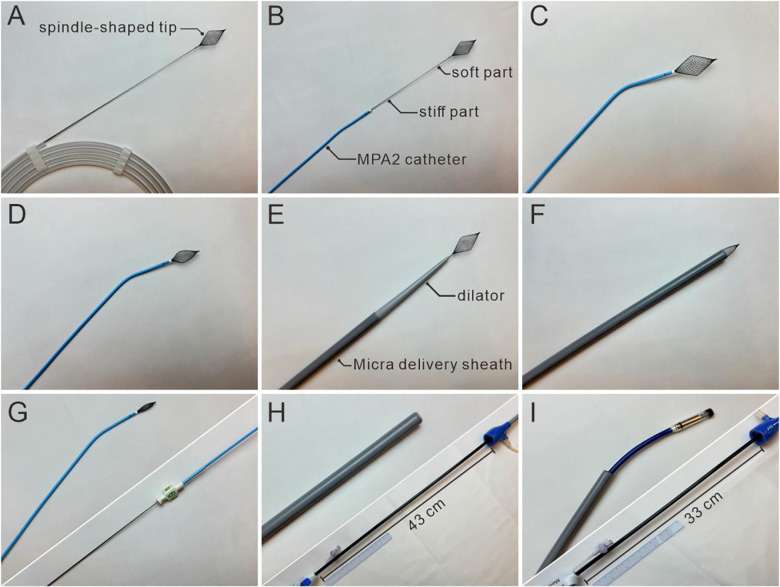
The Panna wire and Micra leadless pacing system. **(A)** Overall display of the wire with its spindle-shaped tip. **(B)** The body of the wire comprises a soft (5 cm) and a stiff part (255 cm). **(C,D)** The wire can be withdrawn into a MPA2 catheter. **(E)** The tip of the wire can not be withdrawn into a dilator of Micra system. **(F)** The wire can be withdrawn into the delivery sheath of Micra system. **(G)** A marker on the wire body has been made during production to fit the length of MPA2. **(H)** Marker (Marker A) should be made manually on the pacemaker delivery system when the tip of pacemaker delivery system aligns with the tip of delivery sheath. **(I)** Another marker (Marker B) should be made manually when pacemaker delivery system advanced 10 cm beyond the delivery sheath which facilitates the system to curve. MPA, multipurpose catheter.

The Panna guidewire (Hangzhou Dexin Medical Technology Co., Ltd., Hangzhou, China) was specifically developed for ultrasound-guided interventional procedures by the Structural Heart Disease Team of Fuwai Hospital. It consists of a rigid main body (stiff shaft, comparable to a conventional super-stiff guidewire) to provide structural support for large-bore sheath delivery, a flexible proximal segment, and a nitinol (nickel-titanium alloy) spindle-shaped tip (Figure 1A). The tip exhibits excellent elasticity and is readily visualized via ultrasound. The guidewire can be withdrawn into a 6-Fr catheter (Figure 1D) and the delivery sheath of the Micra system (Figure 1F), but not into the inner dilator of the Micra delivery sheath (Figure 1E)—a design feature that helps prevent cardiac or venous injury caused by the dilator's sharp tip. Figure 1 depicts the Panna guidewire: a soft, echogenic 5 cm nitinol tip (A) and stiff 255 cm shaft (B). It retracts into MPA2 (C-D) or Micra sheath (F) yet cannot enter Micra's inner dilator (E), averting sharp-tip injury. A factory mark (G) aligns the wire with MPA2; manual ink marks A (flush with sheath tip, H) and B (10 mm distal, I) facilitate controlled curvature across the tricuspid valve (MPA=multipurpose catheter).

### 2.4 Procedure

**Figure 2:**
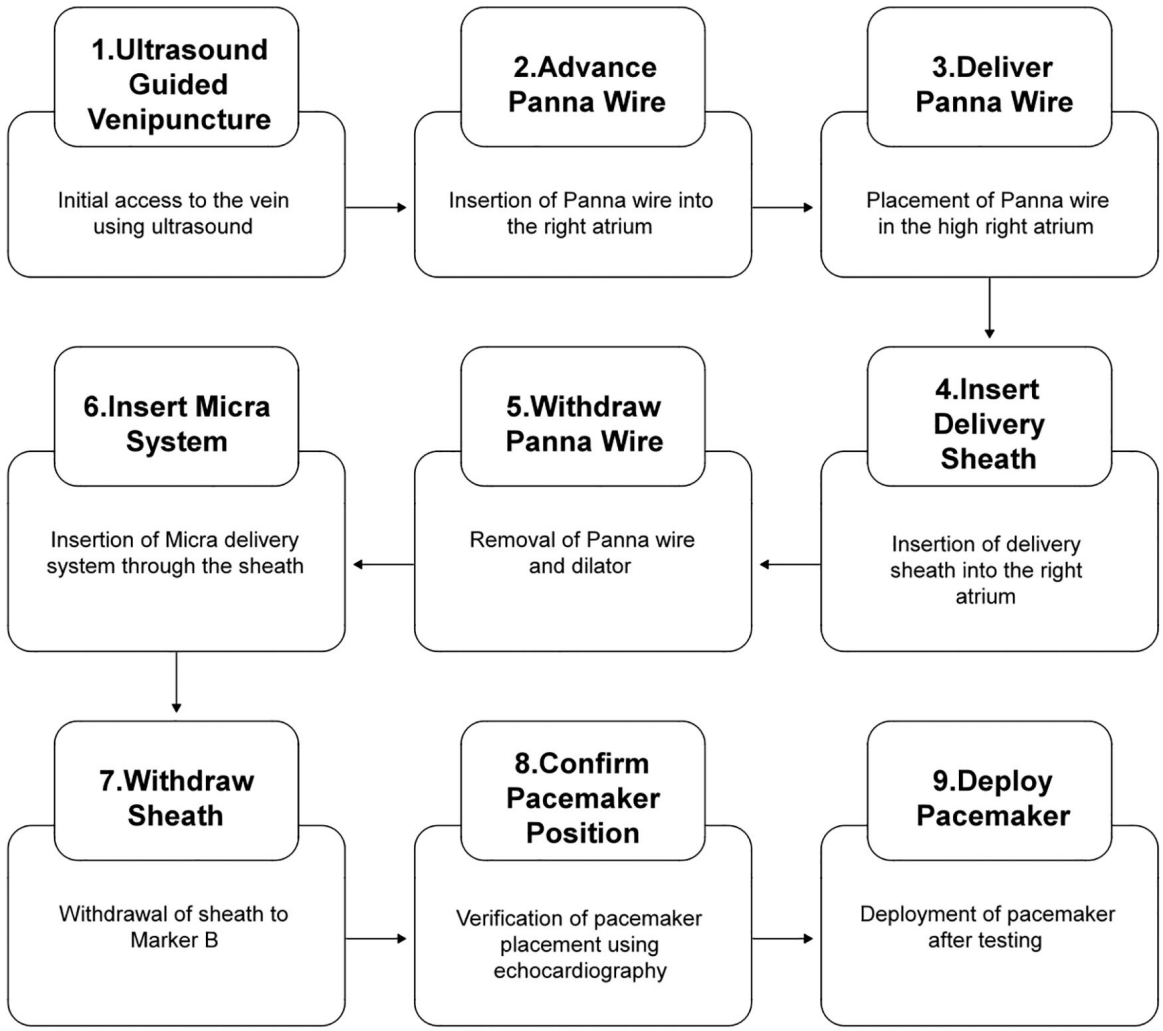
Transthoracic echocardiography guided procedure.

**Figure 3:**
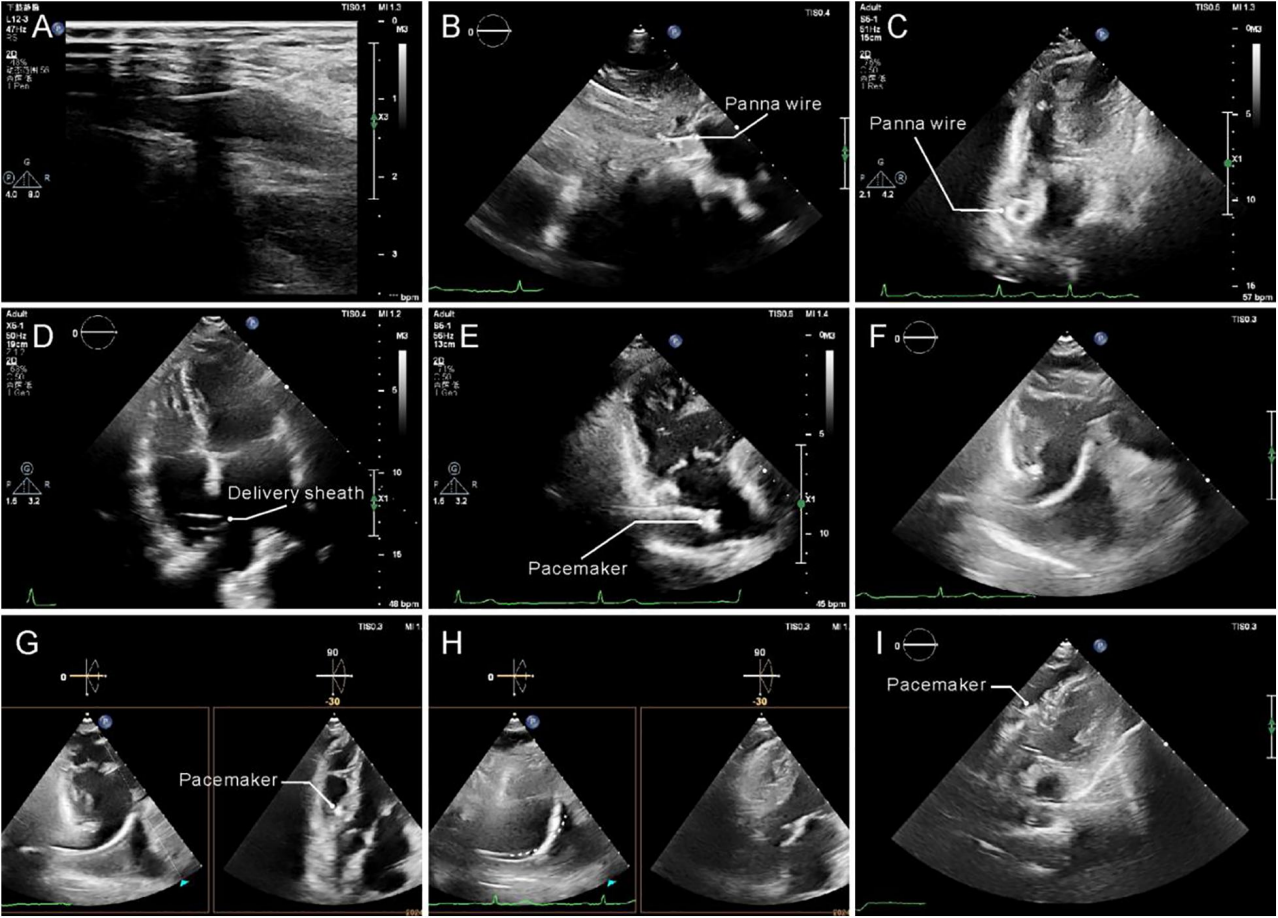
Transthoracic echocardiography guided procedure assisted by Panna wire. **(A)** Ultrasound guided venipuncture. **(B)** Subcostal view shows the advance of Panna wire into the RA, the spindle-shaped tip can be easily detected. **(C)** The Panna wire was delivered to the high right atrium on non-standard trans-apical view. **(D)** After the insertion of delivery sheath, the Panna wire was withdrawn along with the dilator while the delivery sheath was maintained in the high RA position. **(E)** The Micra delivery system was inserted through the sheath until it reached the Marker A, which the tip of pacemaker delivery system align with the tip of delivery sheath. **(F)** The sheath was then withdrawn to Marker B and the Micra system was free to curve across the tricuspid valve. **(G)** The position of pacemaker was confirmed by trans-apical RV inflow view, where as the biplane view shows the pacemaker attached to the ventricular septum without interaction with the RV free wall. **(H)** The Micra system was pushed toward the septum to be held at a high tension position (“gooseneck” sign) from its original position (dash line). **(I)** The pacemaker was deployed after tug test and electrophysiological test. RA, right atrium; RV, right ventricle.

**Figure 4:**
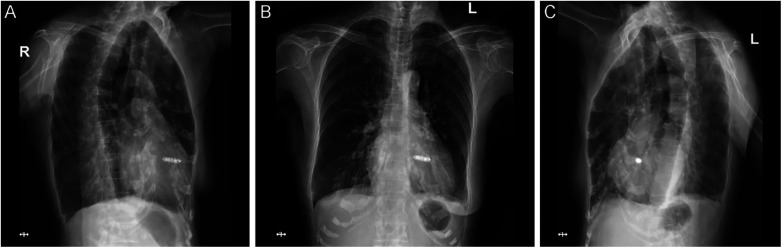
Postoperative chest x-ray at 1-month follow-up showing the stable position of the leadless pacemaker. **(A)** Right anterior oblique view. **(B)** Posteroanterior view. **(C)** Left anterior oblique view.

All procedures were performed under local anesthesia in a cardiac catheterization laboratory, guided by a Philips CVX or EPIC 7C ultrasound system (Philips Healthcare, Amsterdam, Netherlands) with an X5-1 transducer (key steps and imaging are referenced in Figures 2–4). All procedures were completed by a dedicated two-person team: a cardiovascular electrophysiologist (with ≥100 LP implantation experiences and basic TTE proficiency) responsible for interventional operations and real-time coordination, and a specialized cardiovascular ultrasound physician in charge of TTE imaging optimization, real-time structural tracking and positioning feedback. Notably, the Panna guidewire used herein had been previously validated for safety and feasibility in atrial septal defect closure (Kong et al., Circ. Cardiovasc. Interv. 2020), leveraging its ultrasound-optimized spindle-shaped tip to address poor device visibility—a key barrier in TTE-guided interventions. Preoperative TTE acoustic window grading was performed for all patients according to a standardized protocol: Grade 1 (excellent) allowed clear visualization of the right ventricular septum and catheter; Grade 2 (moderate) involved partial obstruction that could be improved by adjusting body position (including left lateral decubitus or forward leaning). Notably, the “forward leaning” maneuver was strictly limited to the pre-operative screening phase to assess feasibility; during the intra-operative procedure, only the supine or partial left lateral decubitus positions were maintained. A Grade 3 (poor) window was defined as the trigger criterion for fluoroscopic backup, and no patients required fluoroscopic conversion in this study.

Pre-procedural TTE was performed to confirm adequate visualization of critical cardiac structures [right atrium (RA), tricuspid valve (TV), right ventricle (RV)], a prerequisite for subsequent TTE guidance. Under direct TTE visualization, the right femoral vein was punctured and a vascular sheath inserted (Figures 2 Step 1, 3A). The Panna guidewire was then loaded into an MPA2 catheter and advanced to the high RA under dual TTE views (subcostal, four-chamber), with its spindle-shaped tip clearly identifiable to confirm correct positioning (Figures 2 Step 2, 3B). Markers on the MPA2 catheter and pacemaker delivery sheath were used to reference the distance from the puncture site to the high RA; two markers on the delivery system (Marker A: sheath-tip alignment; Marker B: 10 cm advancement beyond sheath) facilitated transvalvular manipulation.

The MPA2 catheter was withdrawn while retaining the Panna guidewire in the high RA (Figures 2 Step 3, 3C). After femoral puncture dilation, the delivery sheath (with dilator) was advanced along the guidewire under continuous TTE visualization of the guidewire's spindle tip—critical to avoiding vessel injury (Supplementary Video S11, Figure S2A). Advancement was halted when the guidewire was no longer visible (indicating sheath arrival at the high RA), followed by co-withdrawal of the guidewire and dilator (Figures 2 Step 4, 3D; see also Supplementary Videos S12, S13 and Figure S2B).

The Micra delivery system was inserted to Marker A (Figures 2 Step 5, 3E), then the sheath was retracted to Marker B to allow the system to curve for smooth TV crossing (Figures 2 Step 6, 3F)—monitored via parasternal four-chamber and RV inflow views. To avoid right ventricular outflow tract (RVOT) misplacement (a common fluoroscopy-guided complication), the device remained curved post-TV crossing; gentle rotations facilitated passage past the RV moderator band, with curve release only after confirming the target RV septal landing zone.

Biplane TTE verified that the delivery system avoided contact with the tricuspid valve, chordae tendineae, and RV free wall to prevent valvular damage or arrhythmia induction (Figures 2 Step 7, 3G). This real-time visualization of the tricuspid valve apparatus directly mitigates the risk of mechanical trauma associated with fluoroscopy-only implantation, as fluoroscopy cannot resolve these soft-tissue structures. The Micra system was then advanced to achieve a standardized “gooseneck” sign, verified using strict semi-quantitative criteria: a curvature angle of 90° ± 10° and a contact distance with the RV septum of ≤2 mm. This assessment was performed using a dual-observer consensus protocol (inter-observer consistency Kappa = 0.82) to ensure stable and precise septal contact (Figures 2 Step 8, 3H; Supplementary Video S14, Figure S2C). Subsequently, a standardized TTE-guided tug test was performed: a tugging force of 5–8 N was applied in the direction opposite to catheter delivery for at least 3 s. The success criteria were defined as catheter displacement ≤1 mm under real-time TTE monitoring (demonstrating stable septal apposition in Supplementary Videos S21, S22 and Figure S2D) and no transient changes in pacing parameters, confirming that ≥2 tines were anchored to the RV myocardium. Upon confirmation of fixation stability and satisfactory electrical parameters (sensing, impedance, capture thresholds) pre- and post-test, the tether was cut, and the device was fully released (Figures 2 Step 9, 3I; Supplementary Figure S2E).

Post-procedurally, patients were monitored for 24 h; discharge was permitted only after negative 12-lead ECG and chest x-ray (no acute adverse events: pericardial effusion, device dislodgement, puncture-site infection). All patients were scheduled for standardized face-to-face follow-up at 1, 3, and 6 months post-procedure, which included adverse event screening, arrhythmia symptom assessment, TTE (consistent with intraprocedural views to verify device position), 12-lead ECG for pacemaker function evaluation, and chest x-ray. As of the data cutoff date (May 31, 2025), all 10 patients completed the 1-month follow-up (100% completion rate), 7 patients completed the 3-month follow-up, and 5 patients completed the 6-month follow-up; the difference in follow-up duration was attributed to the sequential enrollment of patients from July 2024 to April 2025. No patients were lost to follow-up, and long-term follow-up is ongoing per the study protocol. No device migration was detected in serial TTE and chest x-ray assessments of all patients at their latest follow-up (Figure 4).

### 2.5 Statistical analysis

All statistical analyses in this study were exploratory and descriptive in nature, with no formal confirmatory non-inferiority hypothesis testing performed given the limited sample size of this proof-of-concept study, and the enrolled sample size of 10 patients was determined based on clinical enrollment feasibility without a confirmatory power calculation for statistical inference. Continuous variables were reported as mean ± standard deviation for normally distributed data (normality verified via the Shapiro–Wilk test) or median (interquartile range, IQR) for non-normally distributed data, while categorical variables were presented as counts with corresponding percentages (%). Propensity score overlap weighting (PSOW) was applied to balance pre-specified baseline covariates between the TTE-guided cohort (*n* = 10) and the historical fluoroscopy-guided control group (*n* = 44, enrolled from January 2020 to December 2023), including age, sex, body mass index (BMI), hypertension, diabetes mellitus, type of bradyarrhythmia, and history of prior transvenous pacemaker implantation, to minimize confounding by indication and temporal treatment bias, with a standardized mean difference (SMD) < 0.1 used to indicate adequate balance of covariates after weighting. Exploratory between-group comparisons were conducted using the independent samples t-test for normally distributed continuous variables, the Mann–Whitney U test for non-normally distributed continuous variables, and the Chi-square test or Fisher's exact test for categorical variables as appropriate. Sensitivity analyses were further performed by comparing the TTE-guided cohort separately with the first 10 consecutive cases and the last 10 consecutive cases of the fluoroscopy-guided group to verify the stability of the exploratory findings and address potential temporal bias associated with the historical control. All statistical analyses were performed using SPSS 26.0 (IBM Corp., Armonk, NY, USA), and a two-sided *P*-value < 0.05 was considered statistically significant for exploratory comparative purposes only, with no adjustment for multiple comparisons performed given the exploratory nature of all analyses.

### 2.6 Patient characteristics

Between July 2024 and July 2025, 10 patients with bradyarrhythmia underwent completely transthoracic echocardiography (TTE)-guided leadless pacemaker (LP) implantation. The cohort had a mean age of 78.20 ± 7.13 years, with four (40%) being female. Comorbidities included hypertension (5 patients, 50%), diabetes mellitus (3 patients, 30%), and a history of ischemic cerebral infarction (1 patient). Additionally, three patients (30%) had a prior transvenous DDD pacemaker with battery depletion. Baseline demographic and clinical characteristics of the enrolled patients are summarized in Table 1.

**Table 1:** Baseline characteristics of 10 patients undergoing TTE-guided LP implantation.

No.	Sex	Age	Diagnose1	Indication class	Duration of disease	BMI	Operation date
CASE1	F	78	III°AVB	Ⅰ	1Y	20.50	2025.01.03
CASE2	F	82	SSS	Ⅰ	3Y	16.20	2024.09.23
CASE3	M	87	SSS	Ⅰ	11Y	18.80	2024.07.02
CASE4	M	87	Af + III°AVB	Ⅰ	1M	21.80	2024.09.24
CASE5	F	68	SSS	Ⅰ	1Y	25.10	2024.08.13
CASE6	M	57	Af + III°AVB	Ⅰ	1Y	28.50	2025.03.09
CASE7	M	79	III°AVB	Ⅰ	1M	17.20	2025.01.09
CASE8	F	84	III°AVB	Ⅰ	4Y	19.90	2025.03.30
CASE9	F	82	SSS	Ⅰ	3Y	22.30	2025.04.07
CASE10	M	73	II°AVB	Ⅱa	1M	14.50	2025.03.22

AVB, atrioventricular block; SSS, sick sinus syndrome; Af, atrial fibrillation; BMI, body mass index; Y, year; M, month.

### 2.7 Safety and feasibility outcomes of LP implantation

All 10 LP implantations were performed in a cardiac catheterization laboratory with fluoroscopy on standby, which was not utilized in any case. Preoperative TTE acoustic window assessment confirmed all patients had Grade 1 (excellent) visibility, consistent with the standardized protocol. Implantation success rate was 100%, with no intraoperative complications (e.g., vascular injury, valvular dysfunction, arrhythmia, or pericardial effusion). The skin-to-skin procedure time—defined as the interval from initial vascular sheath insertion to final sheath removal—averaged 62.78 ± 13.05 min, which was non-significantly longer than that of propensity score-weighted fluoroscopic controls (60.5 ± 19.1 min, *P* > 0.05) with comparable procedural efficiency. Representative intraoperative images of the TTE-guided procedure are shown in Figure 2.

Device selection included Micra VR (5 patients) and Micra AV (5 patients). Regarding implant location: 7 LPs (70%) were positioned on the right ventricular (RV) mid-septum, and 3 (30%) on the RV apical septum; no RV free wall misplacement was observed, confirming the precision of TTE-guided positioning. Only one case required intraoperative LP repositioning due to suboptimal initial pacing thresholds (>1.5 V at 0.24 ms), which was successfully adjusted to satisfactory levels (<0.5 V at 0.24 ms) via biplane TTE guidance. The median postoperative hospital stay was 4 days [interquartile range (IQR), 3–6 days]; all patients met discharge criteria (negative 12-lead ECG, chest x-ray, and TTE with no acute adverse events).

### 2.8 Follow-up outcomes

Over a median follow-up duration of 4.7 months, no device-related complications were observed—including death, LP dislodgement, infection, or pericardial effusion. Postoperative TTE revealed a mean left ventricular ejection fraction (LVEF) of 62.40% ± 5.04%, with no significant decline from baseline (baseline LVEF: 63.12% ± 4.89%, *P* = 0.67). No RV free wall displacement or device migration was detected in serial TTE and chest x-ray assessments (Figure 4).

All 10 patients maintained favorable and stable electrical performance at the last follow-up. Specifically, the median sensing value was 8.2 mV (IQR: 6.5–9.8 mV), median capture threshold was 0.45 V at 0.24 ms (IQR: 0.30–0.60 V), and median impedance was 620 Ω (IQR: 580–660 Ω)—all within the normal reference range for Micra devices. No significant fluctuations in electrical parameters were noted between 1-month and 6-month follow-up, confirming durable device function (Table 2).

**Table 2:** Follow-up results of 10 patients undergoing ultrasound-guided cardiac pacing.

Case	Immediate postoperative			Last follow-up			Follow-up duration	Follow-up date
	Perception (mV)	Threshold (V)	Impedance (Ω)	Perception (mV)	Threshold (V)	Impedance (Ω)	M	
1	9.7	0.6	720	5.8	0.38	650	4.7	2025.05.24
2	7.8	0.3	780	8.8	0.25	720	7.6	2025.05.10
3	0.9	1.5	920	5.4	1.63	890	10.7	2025.05.19
4	12.3	0.3	970	12.4	0.25	920	7.6	2025.05.10
5	4.8	0.3	1,290	17.3	0.38	1,020	9.0	2025.05.10
6	5.1	0.4	1,600	15	0.25	1,000	9.4	2025.05.20
7	10.4	0.3	1,520	13	0.38	890	4.6	2025.05.26
8	5.7	1.5	660	5.8	1.3	630	1.8	2025.05.22
9	9.2	0.5	720	9.0	0.6	690	1.1	2025.05.10
10	6.8	0.6	680	6.5	0.9	650	2.1	2025.05.23

### 2.9 Comparison with fluoroscopy-guided leadless pacemaker (LP) implantation

A historical control group of 44 consecutive patients who underwent fluoroscopy-guided LP implantation at the same institution between January 2020 and December 2023 was included for for exploratory comparative analysis. The control group had a mean age of 77.7 ± 9.8 years, with 22 females (50.0%). Comorbidities included hypertension (*n* = 30, 68.1%), diabetes mellitus (*n* = 5, 11.3%), and a history of transvenous DDD pacemaker battery depletion (*n* = 5, 11.3%). No statistically significant differences in baseline clinical characteristics were observed between the control group and the transthoracic echocardiography (TTE)-guided cohort; detailed demographic and clinical data are presented in Table 3.

**Table 3:** Comparison of baseline characteristics between the two groups of patients [*n*(%), mean ± SD].

Item	TTE-guided (*n* = 10)	Fluoroscopy-guided (*n* = 44)	*p*-value
Female	4 (40.0)	22 (50.0)	0.729
Age (years)	78.2 ± 7.1	77.7 ± 9.8	0.880
BMI (kg·m⁻^2^)	20.5 ± 4.2	23.3 ± 4.0	0.053
Indications for permanent pacemaker implantation			
Af + III° AVB	2 (20.0)	20 (45.4)	0.287
III° AVB, II° AVB	4 (40.0)	19 (43.2)	1.000
SSS	4 (40.0)	18 (41.1)	0.999
Underlying heart disease			
Hypertension	5 (50.0)	30 (68.1)	0.318
Diabetes mellitus	3 (30.0)	5 (11.3)	0.134
Reasons for selecting leadless pacemaker			
Advanced age (≥80 years)	5 (50.0)	21 (47.7)	1.000
Previous transvenous pacemaker battery depletion	3 (30.0)	5 (11.3)	0.134

AVB, atrioventricular block; SSS, sick sinus syndrome; Af, atrial fibrillation; BMI, body mass index.

In the fluoroscopy-guided group, all LPs were successfully implanted at the mid-to-lower right ventricular septum, achieving a 100% procedural success rate. The mean procedural duration was 60.5 ± 19.1 min. Intraoperative electrical parameters were as follows: median R-wave sensing amplitude of 8.2 mV [interquartile range (IQR) 5.9–12.8], median pacing threshold of 0.5 V (IQR 0.4–0.6) at a pulse width of 0.24 ms, and median lead impedance of 770 Ω (IQR 665–970). Procedural complications included 2 cases of small arteriovenous fistula (<3 mm), 1 case of pseudoaneurysm (resolved with timely postoperative compression), and 3 cases of puncture-site venous thrombosis (resolved with anticoagulant therapy). No severe adverse events (e.g., cardiac perforation, pericardial effusion, infection, device dislodgement, or wound dehiscence) occurred. Additionally, one patient underwent postoperative 3.0 T magnetic resonance imaging (MRI) without adverse sequelae.

## 3 Discussion

Leadless pacemakers (LPs) have emerged as a transformative modality for bradyarrhythmia management, with real-world data consistently confirming their superiority over traditional transvenous pacemakers (45, 46)—specifically, 40%–60% lower rates of lead dislodgement, pocket infection, venous thrombosis, and reintervention (13, 14, 47–49). This profile makes LPs uniquely valuable for vulnerable subgroups, such as patients with thin chest walls (at higher risk of pocket complications) or immunocompromised individuals (at elevated infection risk), addressing longstanding unmet needs in these populations (47, 48). However, the clinical potential of LPs has been constrained by their reliance on fluoroscopic guidance—a limitation our study directly addresses by validating a fully transthoracic echocardiography (TTE)-guided approach assisted by the Panna guidewire, with standardized operational protocols (acoustic window grading, semi-quantitative “gooseneck” sign assessment, and TTE-guided tug test) to ensure reproducibility.

### 3.1 Alignment with prior research and technical breakthroughs

It should be noted that the performance of the Micra device itself is well-established by major registries and landmark trials, including the LEADLESS II trial, the 5-year Micra Post-Approval Registry, and the 2021 JAMA Cardiology Micra CED study—all of which have rigorously validated the long-term safety, efficacy, and device performance of Micra LPs in large, multicenter cohorts. Our work focuses on the innovation of the delivery imaging modality rather than re-evaluating the inherent performance or complication rates of the Micra device. Our study's 100% procedural success rate and absence of complications (e.g., peripheral vascular injury, LP dislodgement, pericardial effusion) in 10 patients directly resolves key challenges identified in earlier literature. Notably, all patients had preoperative Grade 1 (excellent) TTE acoustic windows, which, paired with the Panna guidewire's echo-enhancing design, eliminated the “tip loss” risk that plagued prior ultrasound-guided interventions (30, 31, 38). Conventional LP implantation relies on fluoroscopy, exposing vulnerable patients—those with impaired renal function, hematological disorders, or pregnancy—to avoidable harm (50–53). As highlighted by (26), fluoroscopy-only LP implantation is limited by its inability to visualize soft-tissue cardiac structures, including the tricuspid valve, chordae tendineae, and myocardial boundaries, which can lead to subclinical mechanical trauma during sheath advancement and increased risk of valvular dysfunction. Our TTE-guided approach mitigates this risk by providing direct, real-time visualization of the tricuspid valve apparatus and right heart anatomy throughout the procedure, enabling precise manipulation of the delivery system to avoid soft-tissue injury. In contrast, our approach leverages the Panna guidewire's spindle-shaped nitinol tip (readily visualized via 2D TTE) and inherent safety features (non-retractable tip, elastic soft design) to mitigate trauma, while the standardized marking protocol described in Methods streamlines precise positioning. This aligns with our earlier experience using the Panna guidewire for TTE-guided atrial septal defect (ASD) closure (38), confirming its utility as a universal tool for ultrasound-guided structural heart procedures.

A critical advantage of our technique is its anatomical precision: real-time TTE enabled 100% accurate septal deployment (70% mid-septum, 30% apical septum) without RV free-wall placement—resolving the longstanding issue of fluoroscopy-induced mispositioning (20%–30% free-wall rate) linked to ventricular dysfunction (24, 25, 27). Even apical septal positioning, historically high-risk for perforation with fluoroscopy (54–58), was accomplished safely, underscoring TTE's superiority in visualizing soft-tissue boundaries, particularly in patients with variant anatomy. The semi-quantitative “gooseneck” sign (90° ± 10° curvature, ≤2 mm septal contact) and standardized tug test (5–8 N force, ≤1 mm displacement) further ensured stable device anchoring, as reflected by unchanged electrical parameters and no migration during follow-up.

### 3.2 Clinical implications in a broad global context

Beyond technical validation, our results have far-reaching implications for equitable access to high-quality pacing care. At the patient level, fully TTE-guided LP implantation removes barriers for those with fluoroscopy/contrast contraindications (e.g., end-stage renal disease, pregnancy), who historically faced delayed or denied LP therapy (28). Our approach eliminates radiation/contrast risks, allowing these populations to benefit from LP's low complication rates instead of outdated transvenous pacemakers (15, 16). This is supported by our follow-up data: mean LVEF remained stable (62.40% ± 5.04% vs. baseline 63.12% ± 4.89%, *P* = 0.67), and electrical parameters (sensing, capture threshold, impedance) stayed within normal ranges, confirming durable functional outcomes.

At the global level, this technique directly addresses the “fluoroscopy gap” in resource-limited settings—where the scarcity of fluoroscopic equipment is compounded by prohibitive costs of maintenance, radiation protection, and contrast agents (38). Most remote and developing regions lack not only such devices but also the infrastructure to sustain them, leaving patients with only high-complication transvenous pacemakers or no pacing therapy at all, exacerbating morbidity and mortality. Our success in a day-surgery setting—requiring only a standard, portable TTE machine and a trained two-person team (interventionalist + ultrasound physician)—effectively decouples LP therapy from fluoroscopic dependence. This breakthrough enables LP implantation in basic clinical facilities, vastly expanding access to underserved populations and narrowing cardiovascular care disparities between resource-rich and resource-poor regions.

### 3.3 Comparison of safety, procedural efficiency, and clinical benefits between TTE-guided and fluoroscopy-guided endocardial lead implantation

Our TTE-guided cohort achieved 100% procedural success with no perioperative complications, in contrast to the fluoroscopy-guided control group (2 arteriovenous fistulas, 1 pseudoaneurysm, 3 puncture-site venous thrombosis). While all control complications resolved, the difference suggests TTE guidance may mitigate vascular injury risk—attributable to real-time visualization of the Panna guidewire's spindle tip. Additionally, TTE eliminated radiation exposure for patients/operators and CIN risk in renal-insufficient patients, providing a viable alternative for radiation-sensitive groups.

The TTE-guided skin-to-skin procedure time (62.78 ± 13.05 min) was slightly longer but statistically non-inferior to controls (60.5 ± 19.1 min, *P* > 0.05), as confirmed by our non-inferiority analysis. Although ultrasound image acquisition added time, the Panna guidewire's enhanced echogenicity reduced positioning delays, and the elimination of contrast preparation/fluoroscopy calibration balanced overall efficiency. Sensitivity analysis further confirmed the TTE technique's effectiveness was not operator-dependent, addressing concerns about learning curve variability and supporting broader applicability.

### 3.4 Limitations and directions for future research

This study has inherent limitations. First, it is a single-center, retrospective proof-of-concept study with a small sample size (*n* = 10) and short median follow-up (4.7 months), limiting statistical power to detect rare or late adverse events and precluding formal establishment of non-inferiority to fluoroscopy-guided implantation; all statistical comparisons are exploratory and descriptive rather than confirmatory. Second, procedures were performed by a high-volume operator (≥100 LPs), restricting external validity; no learning curve or inter-operator variability analyses were conducted. Third, TTE has technical constraints: measurement variability, suboptimal image quality in patients with concave chest walls or narrow AP diameters (59, 60), and inter-observer variability in subcostal/apical view interpretation—all of which may impact reproducibility. While we used propensity-score overlap weighting to minimize bias in historical controls, these comparisons remain exploratory due to inherent limitations (temporal bias, unmeasured confounders). Fourth, this study focused solely on implantation techniques; currently, there are no dedicated ultrasound-optimized tools for Micra LP retrieval, and any required retrieval would necessitate conversion to fluoroscopy—this represents a key unmet need for the widespread adoption of TTE-guided LP implantation. Fifth, the study only enrolled patients with Grade 1 TTE acoustic windows, limiting the generalizability of the technique to patients with suboptimal acoustic windows (Grade 2/3).

Future research must validate these preliminary findings. Prospective multicenter registries with larger sample sizes (≥50 patients), longer follow-up (≥2 years), and stratification by operator experience and chest wall morphology are needed to confirm long-term safety and broad applicability. Head-to-head prospective controlled trials comparing TTE- vs. fluoroscopy-guided LP implantation, coupled with development of ultrasound-optimized retrieval tools and standardized training protocols for the TTE-guided workflow, are essential before routine clinical adoption. Additionally, studies focusing on patients with suboptimal acoustic windows (Grade 2/3) will help define the technique's full clinical scope.

## 4 Conclusions

This study provides preliminary single-center evidence for Panna guidewire-assisted TTE-guided LP implantation in 10 patients, demonstrating favorable short-term safety and feasibility with comparable efficiency to fluoroscopy-guided implantation. The ultrasound-optimized Panna guidewire and standardized TTE protocols (acoustic window grading, “gooseneck” sign assessment, tug test) ensure procedural reproducibility and positioning precision. Notably, the core innovation of this study is the imaging modality for device delivery—our completely fluoroscopy-free TTE-guided approach does not alter or re-validate the performance of the Micra device, whose safety and efficacy are already well-established by major landmark trials and registries. TTE guidance offers distinct advantages: radiation/contrast-free implantation, expanded access for vulnerable populations (renal dysfunction, pregnancy), precise RV septal positioning to avoid free-wall misplacement, and improved global pacing access in resource-limited settings. However, findings are limited by small sample size, single-center retrospective design, short 4.7-month follow-up, single operator performance and inherent TTE technical constraints, making these conclusions hypothesis-generating only. This exploratory fluoroscopy-free technique requires rigorous validation via large prospective multicenter studies with controlled cohorts, ≥2-year follow-up and learning curve/inter-operator variability assessments before routine clinical use. This work advances fluoroscopy-free pacing development and highlights the translational potential of ultrasound-guided techniques in bradyarrhythmia management.

## Data Availability

The original contributions presented in the study are included in the article/Supplementary Material, further inquiries can be directed to the corresponding author.
