# Supplementary material for: Safety and efficacy of completely transthoracic echocardiography guided leadless pacemaker implantation assisted by Panna guide wire: initial clinical experience
Source: Front Cardiovasc Med. 2026 Mar 26;13:1791724. doi: 10.3389/fcvm.2026.1791724 (PMC13061684; doi:10.3389/fcvm.2026.1791724)
Supplement: Supplementary file 1 [file Datasheet1.pdf]

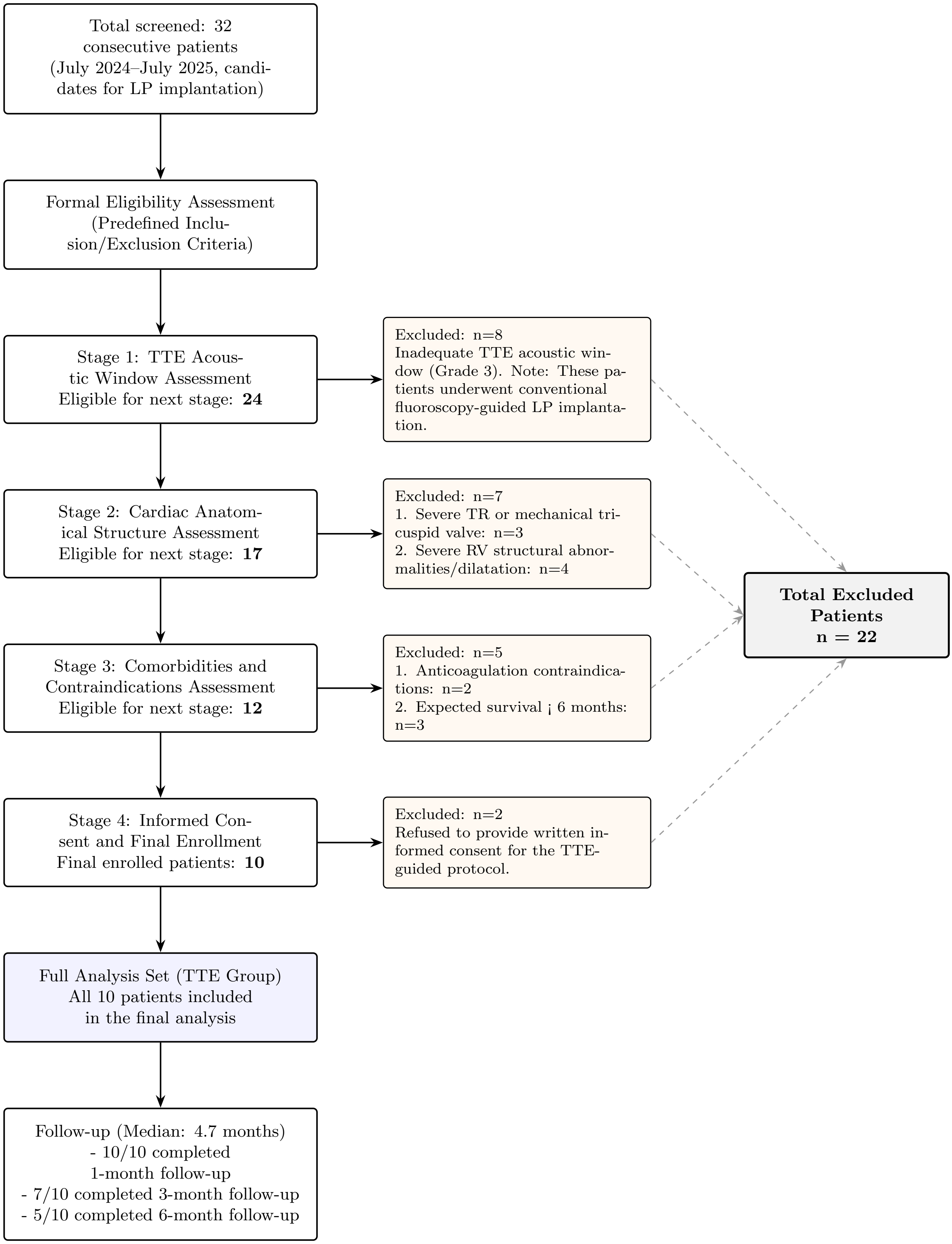

Figure S1: Patient Screening and Enrollment Flowchart.

Note: Grade 3 acoustic window was defined as failure to clearly visualize the right ventricular septum, tricuspid valve, and right atrial anatomy even after optimized body position adjustment.

Abbreviations: LP = leadless pacemaker; TTE = transthoracic echocardiography; TR = tricuspid regurgitation; RV = right ventricular.
