# Supplementary material for: Safety and efficacy of completely transthoracic echocardiography guided leadless pacemaker implantation assisted by Panna guide wire: initial clinical experience
Source: Front Cardiovasc Med. 2026 Mar 26;13:1791724. doi: 10.3389/fcvm.2026.1791724 (PMC13061684; doi:10.3389/fcvm.2026.1791724)
Supplement: Supplementary file 2 [file Datasheet2.pdf]

**Figure S2.** Representative procedural steps and verification of completely TTE-guided leadless pacemaker implantation (Snapshots from Supplementary Videos)

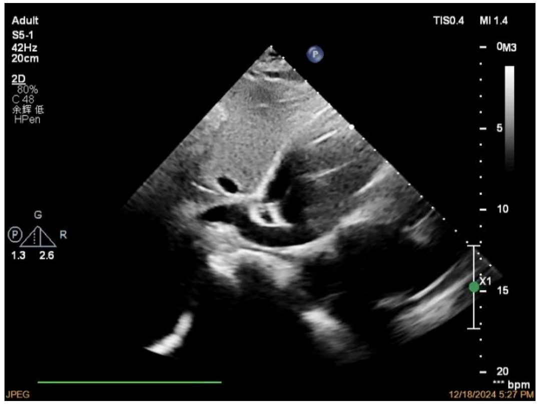

**(A) Panna Guidewire Positioning (Video S11):** The ultrasound-optimized Panna guidewire (rhombic tip) is positioned in the mid-right atrium to measure the distance and guide the subsequent sheath advancement (Addressing **Comment 3**).

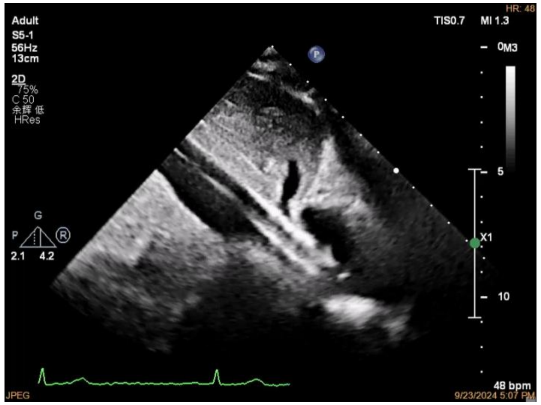

**(B) Sheath Delivery (Video S12-S13):** The delivery sheath advances over the guidewire, visualized as a "double-track sign" in the mid-right atrium, confirming safe tracking without vascular injury (Addressing **Comment 3**).

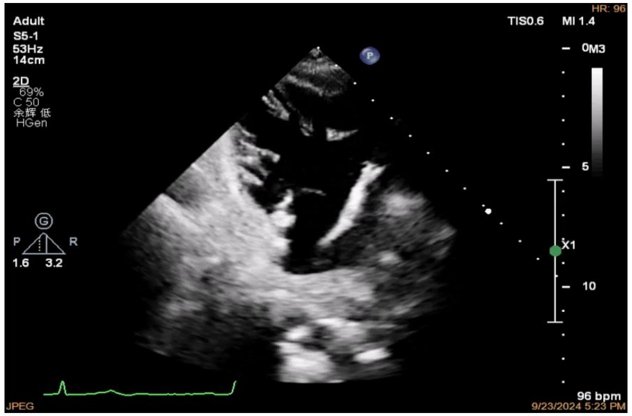

**(C) "Gooseneck" Configuration (Video S14):** The delivery system crosses the tricuspid valve and is articulated into the standardized "Gooseneck" curve at the tip, ensuring perpendicular orientation to the septum (Addressing **Comment 1**).

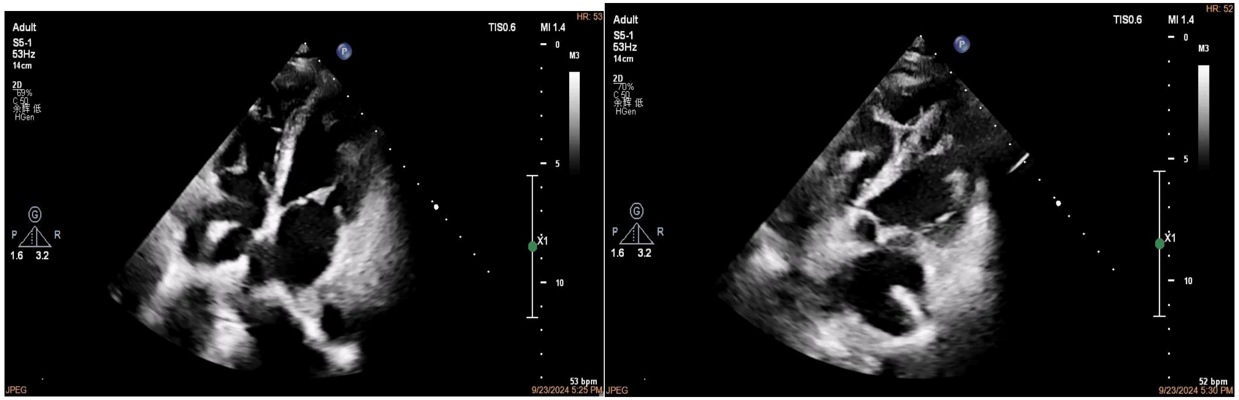

**(D) Septal Contact Verification (Video S21-S22):** Real-time TTE confirms satisfactory apposition of the delivery sheath tip against the right ventricular interventricular septum (IVS) prior to tine deployment.

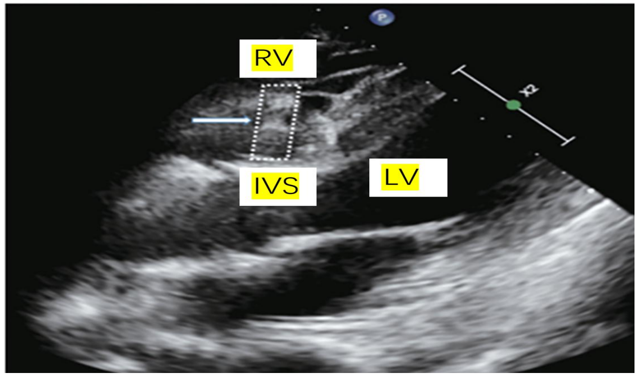


**(E) Final Implantation Verification (Static Image):** The Micra device (white arrow, inside dashed box) is shown securely anchored at the high IVS. This high-resolution static image confirms the stable position achieved immediately following the tug test and tether release (Addressing **Comment 2**).
